# Microbial metabolites as modulators of gut–brain communication in neurodegeneration: from intestinal sensing to disease initiation and progression

**DOI:** 10.3389/fnagi.2026.1926883

**Published:** 2026-10-23

**Authors:** Xi Liu, Sirui Wang, Wei Long, Xinyue Tan, Wanlin Li, LiChen Gao

**Affiliations:** 1 School of Pharmaceutical Science, Department of Pharmacy, Phase I Clinical Trial Centre, The Affiliated Changsha Central Hospital, Hengyang Medical School, University of South China, Changsha, Hunan, China; 2 School of Life Science, Hunan University of Science and Technology, Xiangtan, Hunan, China

**Keywords:** Alzheimer’s disease, epithelial sensing, gut–brain axis, microbial metabolites, Parkinson’s disease, tryptophan metabolism

## Abstract

How heterogeneous gut metabolite alterations relate to Parkinson’s disease (PD) and Alzheimer’s disease (AD) remains unresolved. We propose a framework in which intestinal sensing of indole derivatives, short-chain fatty acids and bile acids links microbial function to barrier regulation and gut–brain communication. Integration denotes tissue-level convergence of aryl hydrocarbon receptor (AhR), pregnane X receptor (PXR) and metabolite-responsive G-protein-coupled receptor responses across epithelial and neighbouring immune cells, rather than an established same-cell receptor complex. Tryptophan metabolism provides an analytical focus connecting microbial indole production with host kynurenine and serotonin pathways; metabolite concentrations alone do not establish pathway flux or receptor activity. We hypothesise that suppressed indole-associated epithelial signalling, barrier hyperpermeability and peripheral immune activation could form a shared gut-derived vulnerability state in subsets of PD and AD. Its co-occurrence and temporal relationship to CNS pathology have not been prospectively demonstrated. Neural, immune and vascular processes may overlap in both diseases, with genotype and cellular context modifying relative susceptibility rather than determining exclusive pathways. Human observational findings are distinguished from experimental mechanisms and proposed interactions. Receptor-directed treatments and metabolite-based biomarkers remain candidates requiring mechanism-specific, safety and clinical validation. Whether a gut-derived intervention window exists, is reversible, or favours earlier treatment requires longitudinal and controlled studies. The framework accommodates bidirectional communication and CNS-intrinsic disease, and generates separate tests of composite gut phenotyping and genotype–metabolite interactions.

## 1 Introduction

Cross-sectional studies associate gut microbial dysbiosis with PD and AD, but its temporal and causal relationship to neurodegeneration remains unresolved (Morais et al., 2021; Tan et al., 2022; Mayer et al., 2022; Cryan et al., 2020; Cryan et al., 2019). Taxonomic shifts vary across cohorts, and composition alone cannot explain how microbial states influence host immune, metabolic or neural responses. Microbial metabolic output offers a complementary functional perspective.

Short-chain fatty acids (SCFAs), secondary bile acid derivatives and tryptophan catabolites engage receptors in intestinal, immune and neural tissues, alongside signals from bacterial structural components and host metabolism (Mayer et al., 2015; Dalile et al., 2019; Nicholson et al., 2012). Metabolite profiles may help explain how compositionally different microbiotas produce related host responses, but such convergence depends on receptor distribution and tissue context and remains to be demonstrated.

For this narrative synthesis, we consider three overlapping perspectives: microbial composition, barrier function and genetic susceptibility. This is an organising scheme rather than an exhaustive classification of previous models. Our proposed contribution is to connect these perspectives through epithelial metabolite sensing and to distinguish established components from untested interactions and disease trajectories.

Epithelial chemosensory and neuroendocrine functions are established in physiological contexts (Bellono et al., 2017), but their coordination in PD/AD remains unresolved. We propose that AhR, PXR and metabolite-responsive GPCR responses across epithelial and neighbouring immune cells jointly influence barrier permeability, mucosal immune tone and enteric–vagal signals (Furness et al., 2013). Integration here denotes tissue-level functional convergence, as developed in Section 3.1; individual receptor functions do not establish a coordinated programme or its therapeutic tractability.

PD and AD studies report altered metabolites and barrier-associated measurements in different populations and sample compartments (Unger et al., 2016; Li et al., 2025; Forsyth et al., 2011; Marizzoni et al., 2025; Qaisar et al., 2023; Chen et al., 2022), without establishing a shared signature or temporal precedence over CNS pathology. The gut-first PD hypothesis proposes an enteric–vagal route for a subset of patients (Borghammer and Van Den Berge, 2019). We examine overlapping neural, immune and vascular mechanisms without assigning a dominant route to either disease.

We hypothesise that disrupted epithelial metabolite sensing could generate a shared gut-derived vulnerability state comprising suppressed indole-AhR/PXR signalling, intestinal hyperpermeability and elevated peripheral immune tone. Their co-occurrence and timing remain unestablished. Tryptophan pathway balance is an analytical focus, while genotype and cellular context are candidate modifiers of downstream susceptibility. Section 4.3 distinguishes this proposed biological state from its measurement proxies; Section 5.2 defines their intended research use.

The framework generates two prospective falsifiable predictions, detailed in Section 6.2.

Reverse causality is plausible: prodromal autonomic dysfunction may alter motility and luminal metabolites (Borghammer, 2018). We conducted a narrative search of PubMed, Scopus and Web of Science for 2010–2025 using ‘gut-brain axis’, ‘microbial metabolites’, ‘tryptophan metabolism’, ‘epithelial sensing’, ‘neurodegeneration’, ‘Parkinson’s disease’ and ‘Alzheimer’s disease’. Selection prioritised mechanistic relevance, human studies and systematic reviews, supplemented by experimental and seminal pre-2010 work. Targeted therapeutic and product/regulatory updates were checked through 24 September 2026; this was not an exhaustive systematic update. No formal PRISMA protocol was applied.

## 2 Microbial metabolites as signalling mediators

### 2.1 Metabolite sources, receptors and disease associations

SCFAs are principally microbial fermentation products; secondary bile acids are host–microbial co-metabolites; and tryptophan catabolites include microbial indoles and host-derived kynurenine and serotonin products. These origins matter when interpreting microbial versus host dysfunction. Faecal metabolite concentrations also depend on diet, absorption and host metabolism, and do not directly measure receptor engagement; selected PD faecal and AD serum findings are discussed below (Unger et al., 2016; Arnold et al., 2020). Metabolomics characterises these small-molecule profiles (Wishart, 2019).

SCFAs—principally propionate and butyrate, which are largely microbially derived, alongside acetate which is additionally produced by host metabolism—represent the most abundant class of gut-derived signalling molecules relevant to neurodegeneration. Beyond serving as energy sources for colonocytes, butyrate has histone deacetylase (HDAC)-inhibitory activity (Davie, 2003), while SCFAs show ligand-specific activity at GPR41, GPR43 and GPR109A (Brown et al., 2003; Kimura et al., 2013; Singh et al., 2014)—the latter a niacin receptor additionally responsive to butyrate that contributes to inflammasome regulation. This dual transcriptional and receptor-mediated action has been proposed to allow SCFAs to preserve epithelial barrier integrity while calibrating the immunological tone of the lamina propria (Parada Venegas et al., 2019; Koh et al., 2016).

Secondary bile acids, including lithocholic acid and deoxycholic acid, are generated by bacterial modification of host primary bile acids. Receptor studies establish distinct mechanisms: bile acids activated TGR5-dependent signalling in receptor-expressing cells and modified macrophage responses (Kawamata et al., 2003), whereas FXR ligand experiments demonstrated transcriptional regulation of bile-acid synthesis and transport (Makishima et al., 1999). These findings do not establish identical potency for every bile acid at both receptors or across all tissues. AD serum studies report associations between bile-acid profiles and cognition (MahmoudianDehkordi et al., 2019). A broader review discusses bile-acid signalling in gastrointestinal inflammation and carcinogenesis (Jia et al., 2018); it does not demonstrate a bile-acid–immune–neurodegeneration sequence. Modulation of neuroinflammatory susceptibility through these receptors is a proposed extension requiring tissue- and ligand-specific testing.

Tryptophan metabolism includes host kynurenine and serotonin pathways and microbial indole-producing routes. Reviews describe the regulation and neuroactive products of the host kynurenine pathway (Cervenka et al., 2017; Schwarcz et al., 2012). We propose that changing pathway activity may influence epithelial and downstream immune or neural responses; direct competition among pathways across different cells and compartments is not established by those reviews. Tryptophan metabolism is therefore used as an analytical focus, rather than as a demonstrated controller of all epithelial signalling. Receptor and pathway experiments derive mainly from cellular and rodent systems unless specified otherwise; human metabolomic observations are identified separately.

Alterations in these metabolite classes have been documented in patient populations, despite the compositional heterogeneity noted in the Introduction. A human PD study measured lower faecal SCFA concentrations than in controls (Unger et al., 2016). A separate microbiota meta-analysis found reduced abundance of several SCFA-producing taxa, which is not equivalent to measuring SCFA concentrations (Nishiwaki et al., 2020). In AD-related cognitive impairment, a small plasma study found lower butyrate but higher acetate and valerate, highlighting differences between metabolite species and sample compartments (Marizzoni et al., 2025). Direct mechanistic evidence linking SCFA alterations specifically to AD pathogenesis in humans remains limited, and findings should be interpreted with caution given this inconsistency. Serum bile-acid profiles were associated with diagnosis, cognition and pathology biomarkers in ADNI analyses (MahmoudianDehkordi et al., 2019; Nho et al., 2019). Separately, DCA and LCA were elevated in a small PD appendix-tissue comparison (Li et al., 2021); these tissue findings should not be treated as equivalent to circulating measurements. In addition, metabolomic analyses in AD cohorts have documented alterations in amino acid and lipid metabolite profiles, providing evidence that systemic metabolic dysregulation extends beyond individual metabolite classes (Arnold et al., 2020). Integrative metabolic network analysis of post-mortem brain tissue from AD patients has revealed altered bile acid synthesis and metabolism, indicating altered brain metabolic pathways without by itself establishing an intestinal origin (Baloni et al., 2020). For tryptophan catabolites, the IPA studies summarised in Table 1 show contrasting directions in AD/MCI and PD, supporting context-specific interpretation rather than a shared decrease (Li et al., 2025; Chen et al., 2022). The framework emphasises epithelial metabolite sensing as an organising principle, but it is important to recognise that several gut-derived signals can exert systemic and central effects through routes that operate in parallel with the epithelial sensing model described here and are not excluded by it. These include: circulating kynurenine, which is predominantly host-derived via IDO1 and TDO2 enzymatic activity rather than microbially produced, and which can undergo carrier-mediated blood–brain barrier transport and supply local synthesis of neuroactive downstream metabolites (Fukui et al., 1991); and lipopolysaccharide (LPS), which is a bacterial structural component rather than a secreted metabolite, and which upon translocation across a permeabilised intestinal barrier activates peripheral pattern recognition receptors (TLR4) to drive systemic inflammatory signalling that indirectly conditions the central neuroimmune environment. The relative contributions of these parallel pathways to neurodegeneration in humans remain to be quantified.

**Table 1:** Human observations and experimental evidence for gut metabolite pathways and intestinal barrier function in PD and AD.

Pathway/endpoint	Evidence design	Population/model	Sample/assay	Measured finding/hypothesis	Supporting reference	Limitation/interpretation
SCFAs	Human—cross-sectional	PD patients vs. age-matched controls (*n* = 34/34)	Faecal samples	Fecal SCFA concentrations significantly reduced in PD vs. controls	Parkinsonism Relat Disord (Unger et al., 2016)	Single time-point, cross-sectional; cannot establish whether reduction precedes or follows disease onset
SCFAs	Human—cross-sectional	CI-AD *n* = 28 (19 MCI/9 dementia); CI-NAD *n* = 29 (19 MCI/10 dementia); CU *n* = 10	Plasma	Lower butyrate but higher acetate/valerate in CI-AD vs. controls (mixed direction across SCFA species)	J Alzheimers Dis (Marizzoni et al., 2025)	Same-cohort AUC: valerate/butyrate 0.918 vs. CU; acetate 0.954 vs. CI-NAD. No independent validation; not prodromal prediction. CIs and intended uses: Section 5.2.
SCFAs	Experimental—mouse/cellular	Wild-type and GPR109A-deficient mice; colonic epithelial and immune-cell assays	Colonic epithelium; macrophages / dendritic cells	GPR109A required for butyrate-induced epithelial IL-18; receptor signalling supported anti-inflammatory immune-cell responses.	Singh et al. (2014)	Specific receptor and inflammation models; does not establish persistent epithelial memory or PD/AD protection.
Indole derivatives (IPA)	Human—cross-sectional	MCI, AD and healthy controls (Fig. 5: *n* = 9–11/group)	Serum and faeces	IPA lower in MCI and AD than controls.	Sci Adv (Li et al., 2025)	Small human comparison within a mixed human/animal study; no PD cohort. Concentrations do not measure receptor activity or establish causality or pre-CNS timing.
Indole derivatives (IPA)	Human—cross-sectional	PD *n* = 56; matched controls *n* = 43	Fasting plasma; LC–MS/MS	IPA higher in PD; no adjusted association with motor or cognitive scores.	J Parkinsons Dis (Chen et al., 2022)	Single-centre, cross-sectional; treated PD cohort; no temporal precedence or direct receptor measurement. Direction differs from AD/MCI; serum and plasma are not interchangeable.
Indole derivatives	Experimental—mouse / cellular	PXR-deficient and PXR/TLR4 double-deficient mice; enterocyte assays	Intestinal barrier; enterocyte inflammatory and junctional readouts	IPA in the context of indole engaged PXR; TLR4 deletion corrected barrier defects in PXR-deficient mice.	Venkatesh et al. (2014)	Ligand and model dependent; no direct PD/AD patient receptor-activity measurement.
Kynurenine pathway	Human—cross-sectional	PD (*n* = 33), AD (*n* = 33), age-matched controls (*n* = 39)	Serum and CSF (paired)	Kynurenic acid reduced in CSF of both PD and AD; kynurenine and quinolinic acid rose with age similarly across all groups	J Neurochem (Sorgdrager et al., 2019)	Age-related increases were similar across PD, AD and controls, confounding disease-specificity; no longitudinal data
Kynurenine pathway	Experimental—human cells	Primary human intestinal epithelial cells and epithelial cell lines	Epithelial IDO1 expression / regulatory signalling	Butyrate reduced IDO1 expression through STAT1-dependent and HDAC-associated mechanisms.	Martin-Gallausiaux et al. (2018)	Expression is not whole-body pathway flux; no PD/AD patient intervention or proof of substrate diversion to microbial indoles.
Secondary bile acids	Human—cross-sectional	PD *n* = 15; controls *n* = 12 (appendix bile-acid analysis)	Appendix tissue; targeted mass spectrometry	DCA and LCA concentrations were higher in PD appendix.	Li et al. (2021)	Tissue concentrations do not establish neuronal exposure or mitochondrial injury; small tissue comparison.
Bile acid profiles	Human—cross-sectional	ADNI *n* = 1,464: CN 370; early MCI 284; late MCI 505; AD 305	Baseline fasting serum; diagnosis / cognition	AD had lower CA and higher DCA; higher DCA: CA associated with worse cognition.	MahmoudianDehkordi et al. (2019)	Baseline association; does not establish gut production, receptor activation or causal direction.
Bile acid profiles	Human—cross-sectional	ADNI *n* = 1,562; biomarker subsets vary	Baseline serum bile acids; CSF Aβ/tau and neuroimaging as outcomes	Serum bile-acid signatures associated with CSF and imaging markers of AD pathology.	Nho et al. (2019)	CSF biomarkers are outcomes, not CSF bile-acid measurements; baseline association cannot establish causal direction.
Bile acids and progression	Human—longitudinal outcome	ADNI MCI *n* = 789; 251 converted, 538 did not within 4 years	Baseline serum bile acids; follow-up AD dementia diagnosis	Lower CA and higher GDCA: CA / TDCA: CA associated with subsequent MCI-to-AD dementia progression.	MahmoudianDehkordi et al. (2019)	Baseline metabolite plus later clinical outcome, not serial metabolomics; MCI may already involve CNS pathology. Does not establish a pre-pathology intervention window.
DCA–*LRRK2* interaction	Proposed/untested	No directly supporting model identified in cited evidence	No measured endpoint	Hypothesis: DCA exposure may modify LRRK2-associated cellular responses; preferential enteric neuronal susceptibility is untested.	Author hypothesis; Section 4.3	Not an empirical finding; factorial genotype × exposure studies and neural/immune comparisons are needed to distinguish interaction, additivity and cell preference.
Epithelial AhR/PXR activity	Mechanistic inference from human observations	MCI/AD and PD cohorts described in IPA rows	Serum / faecal / plasma IPA, not receptor assays	Metabolite differences were measured; epithelial receptor activity was not measured in these cohorts.	Li et al. (2025) and Chen et al. (2022)	Altered metabolite concentration cannot establish reduced receptor activity; directions differ across cohorts.
AhR–immune–epithelial relay	Experimental—mouse	Mouse microbial-indole/AhR studies; epithelial STAT3 deletion in DSS colitis	Mucosal IL-22; epithelial STAT3 and repair	Indole-associated AhR responses supported IL-22; separate experiments linked IL-22-dependent epithelial STAT3 to mucosal repair.	Zelante et al. (2013) and Pickert et al. (2009)	Separate experimental systems; combined receptor integration and human PD/AD effects remain untested.
Intestinal barrier	Human—cross-sectional	Early untreated PD *n* = 9; controls *n* = 10	Oral-sugar urinary recovery; sigmoid biopsies; blood endotoxin-related markers	24-h urinary sucralose increased; lactulose/mannitol ratio unchanged. Permeability correlated with mucosal staining and LBP.	Forsyth et al. (2011)	Small diagnosed-PD cohort; correlated endpoints do not establish metabolite causation or temporal precedence.
Barrier-associated marker	Human—cross-sectional	Mild AD *n* = 71; moderate AD *n* = 66; controls *n* = 74	Plasma zonulin; postural balance testing	AD groups had higher zonulin and poorer balance; zonulin was associated with postural dysfunction.	Qaisar et al. (2023)	Zonulin is an indirect marker, not a functional permeability or tissue assay; association does not prove translocation or causality.
Barrier regulation	Experimental—mice	Anti-CD3-treated wild-type and long-MLCK-deficient mice; jejunal epithelium	MLC phosphorylation; occludin localisation; paracellular BSA flux	Long MLCK deletion or inhibition prevented MLC phosphorylation, junctional disruption and protein leakage.	Clayburgh et al. (2005)	Acute immune activation model; does not test metabolite depletion or PD/AD initiation.

Study design and the inference drawn from a measurement are reported separately. Human cross-sectional rows describe single-assessment comparisons; the human longitudinal row links baseline serum bile acids to later clinical progression, not repeated metabolite measurements. Different analyses within the same cohort are not independent replications. Experimental rows identify the tested species or cellular system and endpoint; their findings do not by themselves establish mechanisms in PD/AD patients. Proposed/untested rows contain author hypotheses, not measured results. Receptor activity, metabolite concentration, functional permeability and indirect barrier-associated markers are distinct measurements. The longitudinal MCI observation provides temporal ordering relative to a later clinical diagnosis, but not relative to the onset of CNS pathology. Prediagnostic symptoms, metabolite observations during a prodromal or MCI stage, and metabolite changes before CNS pathology must not be equated. None of the studies summarised here establishes a metabolite-specific pre-CNS intervention window. Human IPA findings differ between cohorts and sample matrices. ↓, reduced; ↑, elevated; PD, Parkinson’s disease; AD, Alzheimer’s disease; MCI, mild cognitive impairment; SCFA, short-chain fatty acid; IPA, indole-3-propionic acid; IAld, indole-3-aldehyde; DCA, deoxycholic acid; CSF, cerebrospinal fluid; CNS, central nervous system; Kyn/Trp, kynurenine/tryptophan; AhR, aryl hydrocarbon receptor; PXR, pregnane X receptor; HDAC, histone deacetylase; IDO1, indoleamine 2,3-dioxygenase 1; NF-κB, nuclear factor kappa B; IFN-γ, interferon gamma; GPR, G protein-coupled receptor; ADNI, Alzheimer’s Disease Neuroimaging Initiative; CI-AD, cognitive impairment associated with AD; CI-NAD, cognitive impairment not associated with AD. CA, cholic acid; LCA, lithocholic acid; GDCA, glycodeoxycholic acid; TDCA, taurodeoxycholic acid; CN, cognitively normal; LBP, lipopolysaccharide-binding protein; EPEC, enteropathogenic *Escherichia coli*; MLCK, myosin light-chain kinase; MLC, myosin regulatory light chain; DSS, dextran sodium sulfate.

SCFA production and tryptophan catabolism are biochemically distinct but functionally connected within the intestinal environment. Butyrate is predominantly generated through microbial fermentation of dietary substrates; it is not itself a substrate for the tryptophan-to-indole pathway. Their interaction instead includes modulation of host responses to tryptophan-derived signals. In human cell lines and ex vivo human intestinal tissue, Modoux et al. (2022) showed that butyrate enhanced AhR activation by tryptophan-derived agonists through HDAC inhibition and increased accessibility of AhR target-gene promoters. In those experiments, this effect did not require direct binding of butyrate to AhR. Thus, epithelial responses to an indole ligand can depend on the surrounding SCFA environment as well as ligand availability; this finding does not establish an equivalent effect on PXR.

A second connection involves host kynurenine metabolism. Martin-Gallausiaux et al. (2018) found that butyrate reduced epithelial expression of the tryptophan-catabolising enzyme IDO1 in human primary intestinal epithelial cells and cell lines. The reported mechanisms included reduced STAT1 expression, limiting IFN-γ-dependent induction, and a separate STAT1-independent component associated with HDAC inhibition. This provides experimental evidence that a microbial SCFA can regulate a host enzyme controlling tryptophan catabolism. However, reduced epithelial enzyme expression alone does not demonstrate redistribution of tryptophan toward microbial indole production or reduced whole-body kynurenine flux; these outcomes require direct metabolic measurements.

Together, the experiments suggest that butyrate loss could weaken indole-associated AhR responses and reduce restraint on epithelial IDO1 induction. Mouse studies support a role for microbial tryptophan-derived AhR ligands in IL-22 responses (Zelante et al., 2013). The combined SCFA–tryptophan feedback remains an inference from separate experiments, dependent on substrate availability, microbial composition and inflammatory context; neither universal synergy nor temporal precedence over PD/AD is established.

We use tryptophan metabolism as an analytical focus because microbial indole production and host kynurenine/serotonin metabolism provide complementary readouts of host–microbial interactions (Agus et al., 2018; Platten et al., 2019). Their competition within a specific compartment remains to be measured. An indole-to-kynurenine ratio is not a validated readout of integrated AhR/PXR activity, and Kyn/Trp is a different measurement that does not directly quantify microbial–host substrate competition. SCFAs and bile acids remain complementary components of the framework.

### 2.2 Tryptophan metabolism: a competitive signalling hub

Host and microbial enzymes direct tryptophan toward kynurenine, serotonin and indole products. The microbiota can both convert dietary tryptophan and regulate host pathways (Agus et al., 2018). Whether these processes compete for substrate in a particular compartment requires direct flux measurements.

The three main branches of tryptophan catabolism differ sharply in regulatory logic and cellular origin. The kynurenine pathway, responsible for most systemic tryptophan turnover during inflammation, is driven by host enzymes—primarily indoleamine 2,3-dioxygenase 1 (IDO1) and tryptophan 2,3-dioxygenase (TDO)—whose regulation differs by enzyme and tissue; inflammatory induction of IDO1 links immune activity to this pathway, but should not be generalised to identical cytokine regulation of TDO2 (Schwarcz et al., 2012; Platten et al., 2019). In the intestinal context, tryptophan hydroxylase catalyses a rate-limiting step in serotonin synthesis in enterochromaffin cells. Yano et al. (2015) linked microbiota-dependent colonic serotonin biosynthesis to gastrointestinal motility and platelet function in experimental systems; this study does not by itself establish a vagal afferent relay. Microbial tryptophan metabolism generates multiple indole derivatives through distinct enzymatic routes. Lactobacillus-associated IAld production supported mucosal AhR responses in mice (Zelante et al., 2013), while genetic and metabolic experiments identified a reductive aromatic-amino-acid pathway in *Clostridium sporogenes* that includes IPA production (Dodd et al., 2017). IPA, IAld and IAA should not therefore be presented as products of one tryptophanase-catalysed reaction.

Experimental studies support specific links between microbial tryptophan metabolites and mucosal regulation. Indole-associated AhR responses promoted IL-22-dependent mucosal protection in mice (Zelante et al., 2013), whereas IPA in an indole context engaged PXR and altered enterocyte inflammatory and junctional-gene readouts (Venkatesh et al., 2014). Host inflammation can induce IDO1-dependent tryptophan catabolism (Platten et al., 2019; Mellor and Munn, 2004), but whether this reduces substrate availability for microbial indole synthesis in a given intestinal compartment requires direct flux measurements. In CARD9-deficient mice, altered microbiota produced fewer AhR-active tryptophan metabolites, and AhR agonism or selected Lactobacillus strains attenuated colitis (Lamas et al., 2016). These experiments support metabolite–immune interactions, not a demonstrated self-sustaining *IDO1*–indole feedback loop in human PD or AD.

Butyrate-associated HDAC inhibition (Davie, 2003) and enhanced AhR target-promoter accessibility in human experimental systems (Modoux et al., 2022) support metabolite–chromatin coupling. Separate mouse studies establish indole-associated AhR/IL-22 and PXR-dependent barrier responses (Zelante et al., 2013; Venkatesh et al., 2014). They do not demonstrate persistent junctional-gene repression, a specific AhR–KDM6B memory mechanism or failed recovery after metabolite restoration. Persistent epithelial reprogramming remains a hypothesis requiring repeated exposure, withdrawal and recovery studies with locus-specific chromatin and barrier measurements; an epigenetically defined intervention window is not established.

Metabolite concentrations reflect production, absorption and clearance; reduced IPA or elevated kynurenine cannot establish IDO1-mediated substrate diversion (Agus et al., 2018; Platten et al., 2019). An indole-to-kynurenine ratio requires a defined analyte composition and validation and is distinct from Kyn/Trp; neither directly measures epithelial receptor activity.

Table 1 summarises selected human observations and experimental findings relevant to gut metabolite pathways and intestinal barrier function in PD and AD, with study design, sample type, and key limitations specified.

### 2.3 Context-dependent bioactivity of indole derivatives

The biological effects of indole metabolites are highly context-dependent, determined by their receptor affinities, local inflammatory microenvironment and target cell types. Mouse experiments linked IAld-associated AhR responses to IL-22-dependent mucosal protection (Zelante et al., 2013). In separate mouse and enterocyte experiments, IPA in an indole context engaged PXR and altered inflammatory and junctional-gene readouts; TLR4 deletion corrected barrier defects in PXR-deficient mice (Venkatesh et al., 2014). These results support the tested ligand context and PXR–TLR4 mechanism, not a universal epithelial response to IPA. In the AD mouse component of Li et al. (2025), IPA crossed the blood–brain barrier, and neuronal PXR blockade disrupted the reported benefits of intermittent fasting or IPA-related interventions. This supports a model-specific neuronal mechanism, not proof of restoration of human blood–brain barrier integrity or a direct mitochondrial mechanism.

Structurally related indole metabolites can thus trigger distinct and sometimes opposing cellular responses based on receptor context and cell type, as reviewed for AhR signalling (Rothhammer and Quintana, 2019). Whether changing the IAld:IPA balance produces additive or interacting mucosal effects remains to be tested.

Human IPA findings differ in direction between AD/MCI and PD (Li et al., 2025; Chen et al., 2022) (Table 1). These cross-sectional observations establish neither a common depletion signature nor prospective prediction or precedence over CNS pathology.

Experimental AhR/PXR studies support effects of microbial ligands on mucosal responses (Zelante et al., 2013; Venkatesh et al., 2014; Lamas et al., 2016), within a broader catalogue of microbial small molecules (Donia and Fischbach, 2015). Targeting a defined metabolic imbalance is therefore a therapeutic hypothesis requiring disease-relevant validation.

## 3 Intestinal sensing of microbial metabolites

### 3.1 The epithelial sensory Interface: receptor integration

An “integrated epithelial sensory interface” denotes proposed convergence of responses across epithelial subtypes and neighbouring mucosal immune cells on barrier function, cytokines and relay signals (Peterson and Artis, 2014). It does not imply an established same-cell AhR–PXR–GPCR complex. Participating cell populations and receptor combinations remain to be mapped.

An experimentally supported component is communication from mucosal immune cells to the epithelium. In mouse studies, microbial indole-3-aldehyde supported AhR-dependent IL-22 responses and mucosal protection (Zelante et al., 2013). Separately, experiments using intestinal epithelial STAT3 deletion in mice established a role for IL-22-dependent epithelial STAT3 activation in mucosal repair during DSS-induced colitis (Pickert et al., 2009). Together, these studies provide a mechanistic basis for a metabolite–immune–epithelial relay. They do not establish simultaneous integration of AhR, PXR and GPCR signals, or demonstrate this relay as a cause of human PD or AD.

A complementary epithelial pathway is supported by experiments on PXR and TLR4. Venkatesh et al. (2014) found that IPA, in the context of indole, engaged PXR and altered enterocyte inflammatory and junctional-gene readouts; loss of TLR4 corrected barrier defects in PXR-deficient mice. This supports a PXR–TLR4-dependent mechanism in the tested systems, without requiring a direct PXR–AhR interaction. GPCR-mediated sensing provides another component: in mouse experiments, GPR109A was required for butyrate-induced epithelial IL-18 and supported anti-inflammatory functions of colonic macrophages and dendritic cells (Singh et al., 2014). This specific evidence should not be generalised to every SCFA- or bile-acid-responsive GPCR.

We propose that these pathways can functionally converge through cytokine-dependent regulation of epithelial repair and inflammatory stress: immune-cell AhR signalling may influence IL-22–STAT3-mediated epithelial repair, while epithelial PXR–TLR4 regulation and GPR109A-dependent epithelial and immune responses may alter the inflammatory conditions in which repair occurs. This combined mechanism is an inference from the separate experimental pathways above, not demonstrated three-receptor synergy. NF-κB is a candidate downstream inflammatory convergence point for further testing; a shared AhR/PXR/GPCR transcriptional programme in intestinal epithelial cells has not been established by the studies cited here. A same-cell integration model would additionally require evidence of receptor co-expression and functional activity in a defined epithelial subtype, intestinal region and ligand context. Factorial ligand exposure with receptor-specific perturbation in organoids and epithelial–immune cocultures could distinguish independent, additive and interacting responses using permeability, cytokine and transcriptional readouts. Figure 1 illustrates the tryptophan–AhR/PXR axis; it should not be interpreted as proof of same-cell receptor integration. Human IPA measurements do not establish epithelial AhR/PXR activity (Li et al., 2025; Chen et al., 2022). Local ligand exposure, receptor activation and barrier function must be measured separately before a patient-level mechanism can be inferred.

**Figure 1:**
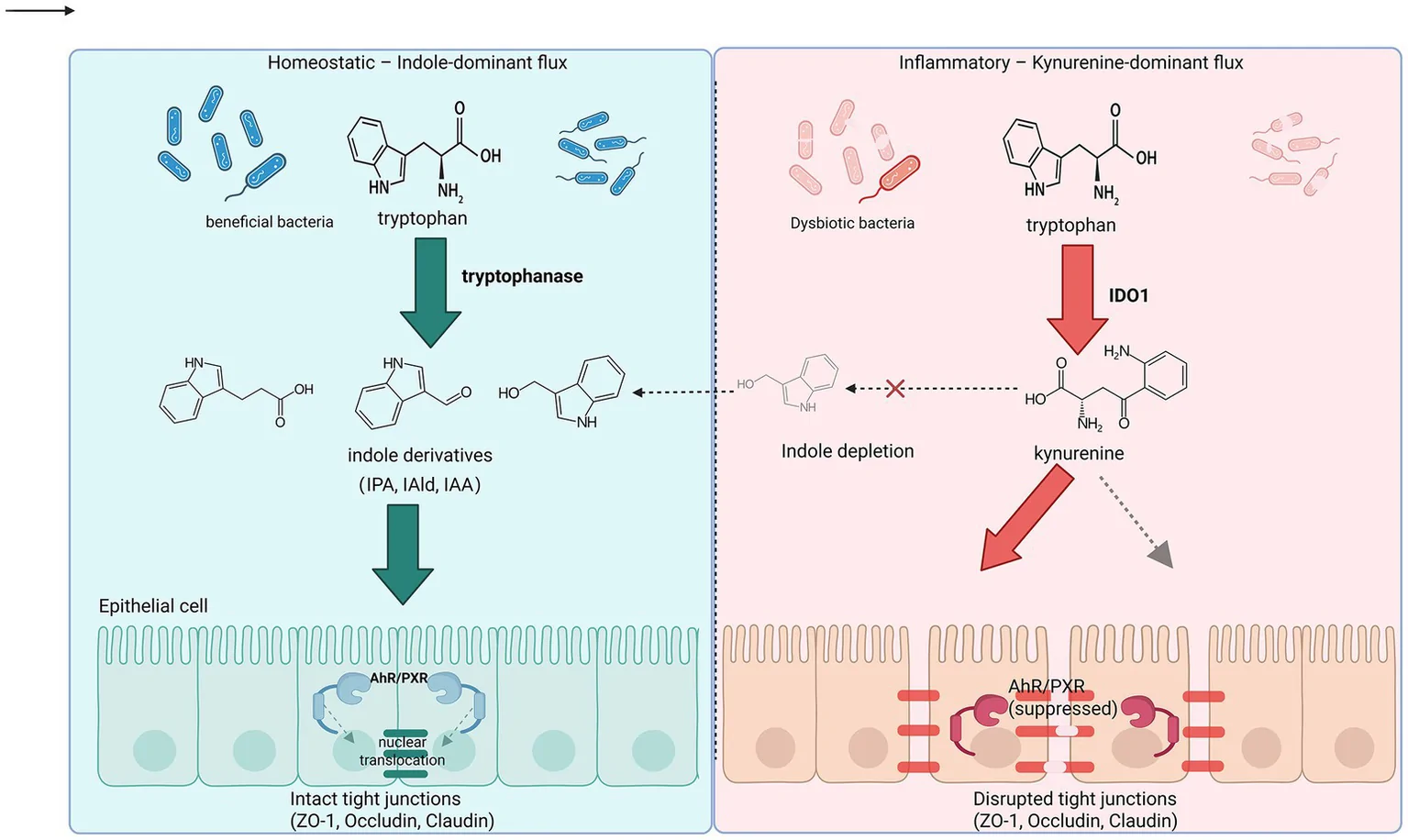
Tryptophan metabolism and epithelial sensing in intestinal barrier regulation. The schematic contrasts a homeostatic, indole-associated state (left, blue) with an inflammatory, kynurenine-associated state (right, pink). Left: microbial tryptophan metabolism supplies indole derivatives that can engage epithelial AhR and PXR signalling and support tight junction maintenance. The indole branch summarises several microbial biosynthetic routes rather than a single tryptophanase-catalysed reaction. Right: increased host kynurenine-pathway activity is depicted alongside reduced indole availability and impaired epithelial junctions. Reduced indole-associated signalling may weaken barrier-supporting transcriptional responses; receptor effects depend on the ligand and cellular context. Coloured arrows indicate schematic metabolic or signalling direction. The crossed dotted connection represents proposed imbalance between tryptophan pathways, rather than conversion of kynurenine into indoles. Nuclear signalling and junctional changes illustrate the proposed link between metabolite sensing and barrier function. The two panels represent conceptual states, not a consistent patient metabolite signature or an established temporal sequence. Measured metabolite concentrations do not demonstrate receptor activity, persistent epithelial memory or a reversible pre-CNS intervention stage. AhR, aryl hydrocarbon receptor; PXR, pregnane X receptor; IDO1, indoleamine 2,3-dioxygenase 1; IPA, indole-3-propionic acid; IAld, indole-3-aldehyde; IAA, indole-3-acetic acid; ZO-1, zonula occludens-1. Created with BioRender.com (Agreement number: HJ29NLJ4NA; https://BioRender.com/bl4ls8u).

### 3.2 Barrier dysfunction and systemic translocation of gut-derived signals

Intestinal permeability is not a fixed structural property of the epithelium, but a dynamically regulated state that determines which luminal signals gain access to the systemic circulation. Clinical permeability observations in early untreated PD do not by themselves establish that changing metabolite exposure causes barrier dysfunction (Forsyth et al., 2011). Under homeostatic conditions, tight junction complexes—composed of claudins, occludin and zonula occludens proteins including ZO-1—maintain selective paracellular sealing, while metabolite–receptor signalling actively supports their expression (Venkatesh et al., 2014; Turner, 2009). This coupling between metabolite sensing and tight junction regulation suggests that permeability may act as a downstream output of the epithelial sensory system, not an independent structural variable.

Several experimental mechanisms could contribute to barrier dysfunction, but their activation by a kynurenine-dominant profile has not been established. PXR-deficient mice showed barrier defects linked to TLR4 signalling, while IPA-associated responses included altered enterocyte TNF-α and junctional mRNAs (Venkatesh et al., 2014). Cytokines can alter junctional expression, localisation and permeability through multiple pathways (Capaldo and Nusrat, 2009). In an anti-CD3 mouse model, long MLCK deletion or inhibition prevented increased epithelial MLC phosphorylation, tight-junction disruption and paracellular protein leakage (Clayburgh et al., 2005). These separate experimental mechanisms motivate a proposed feed-forward cycle: as barrier function declines, luminal bacterial structural components such as lipopolysaccharide (LPS) access the lamina propria, activating pattern-recognition receptors on resident macrophages and dendritic cells. These cells in turn amplify cytokine production and further destabilise tight junction integrity (Guo et al., 2015; Abreu, 2010).

Paracellular permeability and transcellular uptake are distinct routes; their relative importance cannot be ranked uniformly for LPS, other bacterial components and chemically diverse metabolites. Endocytic pathways in enterocytes and M cells in Peyer’s patches provide routes by which intact bacteria and larger molecular complexes can cross the epithelium (Neutra and Kozlowski, 2006). The relative contribution of each pathway likely depends on disease stage and intestinal region. Whether transcellular transport contributes directly to PD or AD pathogenesis—or merely facilitates bacterial sampling under homeostatic conditions—remains to be determined.

Human studies measure different barrier endpoints: early untreated PD showed increased urinary sucralose recovery but unchanged lactulose/mannitol, alongside mucosal and blood-marker associations (Forsyth et al., 2011); AD plasma zonulin was associated with postural dysfunction, without a functional permeability assay (Qaisar et al., 2023). Neither establishes metabolite-driven barrier failure or a neurodegeneration-specific threshold. Barrier dysfunction could alter systemic exposure to bacterial components, while metabolite exposure also depends on transport and clearance. In germ-free mice, increased blood–brain barrier permeability improved after sodium butyrate treatment or colonisation with SCFA-producing bacteria (Braniste et al., 2014). This supports experimental microbiota–neurovascular regulation, not the complete gut-to-CNS sequence in human PD/AD (Braniste et al., 2014).

### 3.3 Enteroendocrine cells and rapid neural relay

Enteroendocrine neuropod cells transmitted luminal signals to vagal neurons through millisecond glutamatergic synapses in mice (Kaelberer et al., 2018). This differs from endocrine or neural effects of hormones such as GLP-1 (Gribble and Reimann, 2019); hormone release should not be equated with the same rapid synaptic response.

Neuropod cells form synaptic contacts through basal processes, but direct confirmation of this architecture in human intestine remains limited (Kaelberer et al., 2018; Bohórquez et al., 2015).

We hypothesise that altered SCFA and indole exposure modifies enteroendocrine signalling and enteric–vagal activity. Anatomical continuity with brainstem circuits motivates this possibility without proving metabolite-triggered α-synuclein pathology (Zhang et al., 2023; Seguella and Gulbransen, 2021). Rapid synaptic, slower endocrine and immune responses operate on overlapping timescales; their relative contributions to PD/AD and the duration of any persistent epithelial response remain unknown. These are general communication mechanisms, and the complete metabolite–neuropod–neurodegeneration sequence has not been demonstrated in patients.

## 4 Signal relay: from intestinal dysfunction to central vulnerability

Gut metabolite disturbances may influence CNS vulnerability through overlapping neural, immune and vascular routes (Figure 2). The PD and AD examples below do not imply exclusive disease–pathway assignments. Bidirectional gut–brain communication provides the broader context (Fung et al., 2017; Needham et al., 2020); metabolite manipulation affecting mouse brain activity and anxiety-related behaviour offers adjacent evidence, not a PD/AD model (Needham et al., 2022).

**Figure 2:**
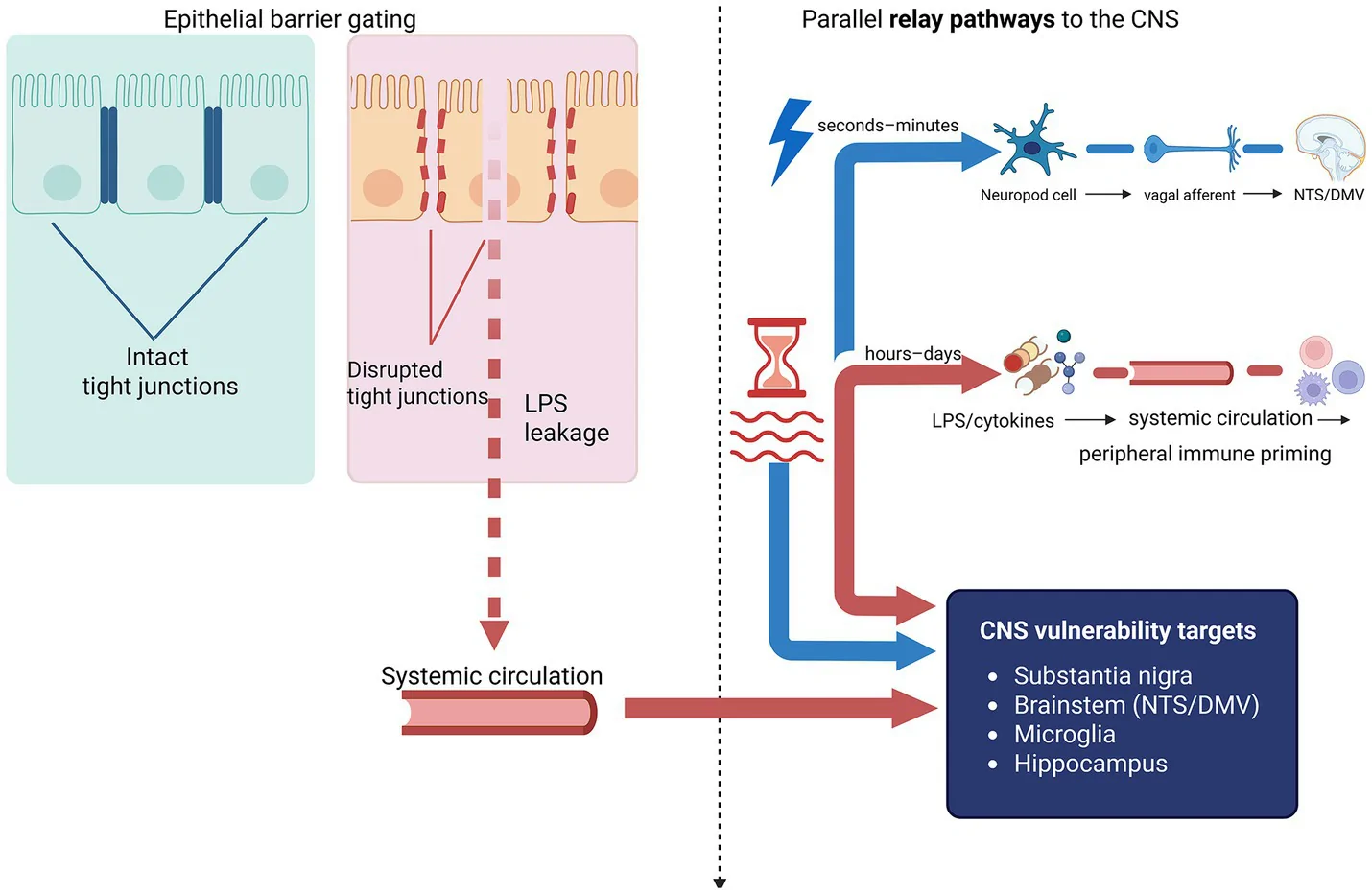
Epithelial barrier gating and parallel neural and immune routes of gut–brain communication. Left: intact tight junctions restrict paracellular passage, whereas junctional disruption can facilitate translocation of luminal bacterial components. The dashed red arrow depicts LPS leakage toward the systemic circulation; LPS is a bacterial structural component. Right: the blue route represents sensory transmission from neuropod cells through vagal afferents to brainstem nuclei. The red route represents circulating bacterial components and cytokines, peripheral immune activation, and potential downstream effects on CNS targets. The “seconds–minutes” and “hours–days” labels schematically contrast rapid neural signalling with slower immune responses, rather than specifying the timescale of neurodegeneration. The lower box lists candidate brain regions and cellular targets. Solid coloured arrows indicate the direction of communication, and the vertical dashed line separates the two panels. Neural and immune routes may interact and contribute to both PD and AD; the complete gut-to-CNS disease sequence remains a proposed framework. LPS, lipopolysaccharide; CNS, central nervous system; NTS, nucleus tractus solitarius; DMV, dorsal motor nucleus of the vagus; PD, Parkinson’s disease; AD, Alzheimer’s disease. Created with BioRender.com (Agreement number: QD29NLJ7SJ; https://BioRender.com/l6fui0g).

### 4.1 The neural relay route: PD and enteric α-synuclein biology

In a proposed gut-first PD trajectory, enteric α-synuclein pathology could propagate through vagal connections. Braak staging describes distributions of Lewy pathology in post-mortem brain tissue (Braak et al., 2003); it does not directly track gut-to-brain progression or establish the duration of a premotor interval within individuals. Alternative pathological distributions, including limbic-predominant cases, limit the universality of a single ascending sequence (Beach et al., 2009). Brain-first and gut-first models may describe different PD subgroups (Borghammer and Van Den Berge, 2019; Borghammer, 2018). In brain-first disease, gut dysfunction and altered metabolites could be consequences or amplifiers of CNS pathology. A three-case study detected colonic α-synuclein immunostaining in biopsies obtained 2 to 5 years before motor onset (Shannon et al., 2012), but this neither established earlier metabolite changes nor excluded concurrent CNS pathology. The framework proposed here is most directly relevant to testing gut-first mechanisms. Experimental models and human observations are distinguished below.

Several separate findings motivate, but do not establish, a metabolite-dependent enteric vulnerability mechanism. Butyrate inhibits HDAC activity (Davie, 2003), and sodium butyrate improved inflammatory and neuronal readouts in an MPTP mouse model (Guo et al., 2023); neither result proves that endogenous butyrate withdrawal initiates enteric α-synuclein pathology. Enteric glia participate in local inflammatory and neuronal responses (Seguella and Gulbransen, 2021), but a specific SCFA-withdrawal–gliosis sequence requires direct experimental support. In α-synuclein-overexpressing mice, colonisation with curli-producing bacteria worsened α-synuclein pathology and behavioural deficits, and purified curli accelerated aggregation in biochemical assays (Sampson et al., 2020). This is evidence for a bacterial structural amyloid, not an indole or SCFA mechanism. We hypothesise that altered metabolite exposure could modify neuronal oxidative stress and protein clearance; an enteric AhR–antioxidant or butyrate–HDAC–TFEB chain remains to be demonstrated in the same defined model.

Prior vagotomy reduced gut-to-brain α-synuclein transmission in rodents, supporting dependence on neural connectivity (Kim et al., 2019). Human truncal-vagotomy data suggested reduced subsequent PD risk (Svensson et al., 2015); a Swedish cohort found no overall association, with lower risk confined to some truncal-vagotomy analyses (Liu et al., 2017). Residual confounding, variation by surgical subtype and limited statistical power make these associations hypothesis-generating rather than confirmatory (Svensson et al., 2015; Liu et al., 2017).

Experimental α-synuclein studies support distinct innate immune responses. Specific extracellular oligomeric conformations released from neuronal cells activated microglial TLR2 (Kim et al., 2013). Separately, primary wild-type and TLR4-deficient mouse microglial and astroglial cultures were exposed to soluble, fibrillised or C-terminally truncated α-synuclein; TLR4 influenced inflammatory, oxidative and phagocytic responses, with cell- and preparation-dependent effects (Fellner et al., 2013). This is not equivalent evidence that the same oligomer is a direct ligand for both receptors. Neither study establishes a preceding intestinal metabolite disturbance or the full transition from peripheral vulnerability to persistent CNS pathology.

We hypothesise that barrier-associated immune signalling could modify microglial responses alongside neural propagation. A metabolite-dependent reduction of the substantia nigra inflammatory threshold has not been demonstrated by the studies cited here. Experimental gut-to-brain propagation (Kim et al., 2019) and microbiota-dependent motor and inflammatory effects in α-synuclein-overexpressing mice (Sampson et al., 2016) support different components; neither establishes a dominant disease-modifying route across human PD.

### 4.2 The systemic immune relay route: AD and microglial priming

Systemic inflammation is a candidate contributor to AD (Perry et al., 2007; Wyss-Coray and Rogers, 2012); immune-to-brain studies in sickness behaviour and depression provide broader context (Dantzer et al., 2008). Germ-free APP-transgenic mice had reduced Aβ burden, which increased following colonisation with microbiota from conventionally raised APP-transgenic mice (Harach et al., 2017). Separately, human bacterial-taxa and inflammatory-marker associations with amyloidosis were observational (Cattaneo et al., 2017). These findings motivate testing gut-associated immune conditioning alongside neural and vascular processes, without establishing its dominance or a gut-dependent threshold for AD progression.

Microglia, the brain’s resident innate immune cells, are highly responsive to peripheral inflammatory signals and can shift to a sensitised activation state following prolonged exposure to circulating cytokines and metabolic mediators. Microglial priming provides a candidate mechanism for this proposed connection; its gut-dependent initiation in human AD has not been established (Heneka et al., 2015; Leng and Edison, 2021; Salter and Stevens, 2017). Germ-free, microbiota-depletion and recolonisation experiments in mice demonstrate effects on microglial maturation and homeostasis (Erny et al., 2015). A developmental review discusses microglia–neuron interactions and peripheral signals during circuit formation (Thion et al., 2018). Neither establishes gut-induced microglial priming as a causal sequence in human AD. Microglial responses to amyloid depend on receptor and cellular context (Heneka et al., 2015; Leng and Edison, 2021). Experiments showed that oligomeric Aβ bound TREM2 and promoted TREM2-dependent signalling, migration and clustering (Zhong et al., 2018); this study alone does not demonstrate a gut-conditioned priming sequence. A review of disease-associated microglia (DAM) synthesises single-cell and genetic findings and proposes a damage-sensing role for these cells; it does not establish a gut-induced response shared uniformly across neurodegenerative diseases (Deczkowska et al., 2018).

Circulating cytokines may influence microglia without immune-cell entry into the CNS (Heneka et al., 2015; Varatharaj and Galea, 2017). Human *APOE* ε4-associated barrier abnormalities and prediction of cognitive decline by a pericyte-injury marker (Montagne et al., 2020) are distinct from general barrier reviews (Banks, 2016; Sweeney et al., 2018). Candidate routes include circumventricular access, endothelial transport and endothelial signalling (Varatharaj and Galea, 2017; Sweeney et al., 2018). A specific gut-metabolite signature causing persistent microglial priming in patients remains unestablished.

Peripheral kynurenine metabolism may influence the CNS through precursor transport and subsequent local metabolism, which should be distinguished from direct entry of downstream metabolites. In an *in situ* rat brain-perfusion study, circulating L-kynurenine crossed the blood–brain barrier through a saturable large neutral amino acid transport system (L-system), rather than by virtue of lipophilicity (Fukui et al., 1991). In the same study, kynurenic acid (KYNA) and quinolinic acid (QUIN) showed much lower permeability and were not expected to contribute substantially to brain pools under normal conditions (Fukui et al., 1991). Thus, peripheral KYNA or QUIN concentrations cannot be treated as direct measures of CNS exposure. Within the CNS, kynurenine can supply local KYNA and QUIN synthesis, depending on cellular enzyme expression and inflammatory context. Cytokine-stimulated human astrocyte cultures produced kynurenine and KYNA with little net QUIN accumulation, whereas macrophage cultures converted kynurenine to QUIN (Guillemin et al., 2001). These cell-culture findings support cell-dependent metabolism, not a fixed glial-versus-neuronal division or demonstrated pathway flux in patients with PD or AD. The balance of local synthesis, degradation and transport therefore needs to be considered when interpreting circulating kynurenine-pathway measurements.

Bile-acid associations do not establish TGR5/FXR activity or subsequent microglial priming. Lower CSF 7α,25-dihydroxy-3-oxocholest-4-en-26-oic acid in AD (Griffiths et al., 2019) does not establish intestinal origin or CNS transport of microbial secondary bile acids. Serum findings motivate receptor-specific testing alongside established AD pathology (MahmoudianDehkordi et al., 2019; Nho et al., 2019). DCA-induced NLRP3-related macrophage responses and worsened DSS colitis in mice (Zhao et al., 2016) provide an inflammatory mechanism whose extension to AD microglial priming is untested.

### 4.3 A shared gut-derived vulnerability state with divergent downstream trajectories

The proposed shared state is not confirmed by current human findings. IPA directions differ between AD/MCI and PD (Li et al., 2025; Chen et al., 2022); paired serum/CSF kynurenine studies do not establish disease-specific IDO1 activation (Sorgdrager et al., 2019); and PD faecal SCFA reductions (Unger et al., 2016) cannot be equated with mixed AD-related plasma changes (Marizzoni et al., 2025). AD microbiota composition (Zhuang et al., 2018) is not a metabolite measurement. Abnormal microbial metabolism in IBD (Lamas et al., 2016; Franzosa et al., 2019) and impaired AhR-ligand production in metabolic syndrome (Natividad et al., 2018) also challenge specificity without establishing identical signatures. No cited study demonstrates co-occurrence of suppressed epithelial indole-AhR/PXR signalling, hyperpermeability and elevated peripheral immune tone before CNS pathology in PD/AD. Candidate proxies include IPA, a defined indole-to-kynurenine ratio, cytokines, LBP and functional oral-sugar permeability measurements. None alone confirms receptor activity, pathway flux or intestinal origin. Early PD showed increased sucralose recovery, unchanged lactulose/mannitol and lower LBP (Forsyth et al., 2011), precluding an assumed elevated-LBP rule. Derivation from established-disease observations risks circular validation. Independent tests must separate improved gut phenotyping, genotype–metabolite interaction and temporal precedence; diagnostic thresholds or PD-versus-AD performance cannot be assumed (Sections 5.2 and 6.2).

The state is not proposed as a necessary or specific condition for neurodegeneration; related abnormalities occur in IBD and metabolic syndrome (Lamas et al., 2016; Natividad et al., 2018). Chronicity, ageing, genotype and cellular context could modify its consequences. A combined indole/secondary-bile-acid disturbance is one testable candidate, but DCA-induced mitochondrial membrane damage in enteric neurons and direct IPA inhibition of α-synuclein fibrillation remain unproven. Defined enteric-neuron exposure studies and cell-free aggregation assays must distinguish these mechanisms from general antioxidant or cytoprotective effects. Enrichment of this combination in PD relative to IBD or healthy ageing, and its timing relative to CNS pathology, also require testing.

Unresolved questions concern co-occurrence, disease specificity, causal direction and patient-level receptor measurements. Tryptophan metabolism remains an analytical focus rather than a demonstrated master regulator; the distinct temporal tests are discussed in Section 5.3.

Genetic background may modify the relative susceptibility of overlapping neural, immune and vascular processes, rather than assign *LRRK2/GBA1* variant carriers to a neural PD pathway and *APOE* ε4 carriers to an immune AD pathway. Existing gut and glial literature motivates these candidate modifiers (de Guilhem de Lataillade et al., 2023; Fernandez et al., 2019), but does not establish preferential responses to a shared metabolite disturbance. Figure 3 should therefore be read as a hypothesis about relative pathway susceptibility, not a genetic separation of PD and AD relays.

**Figure 3:**
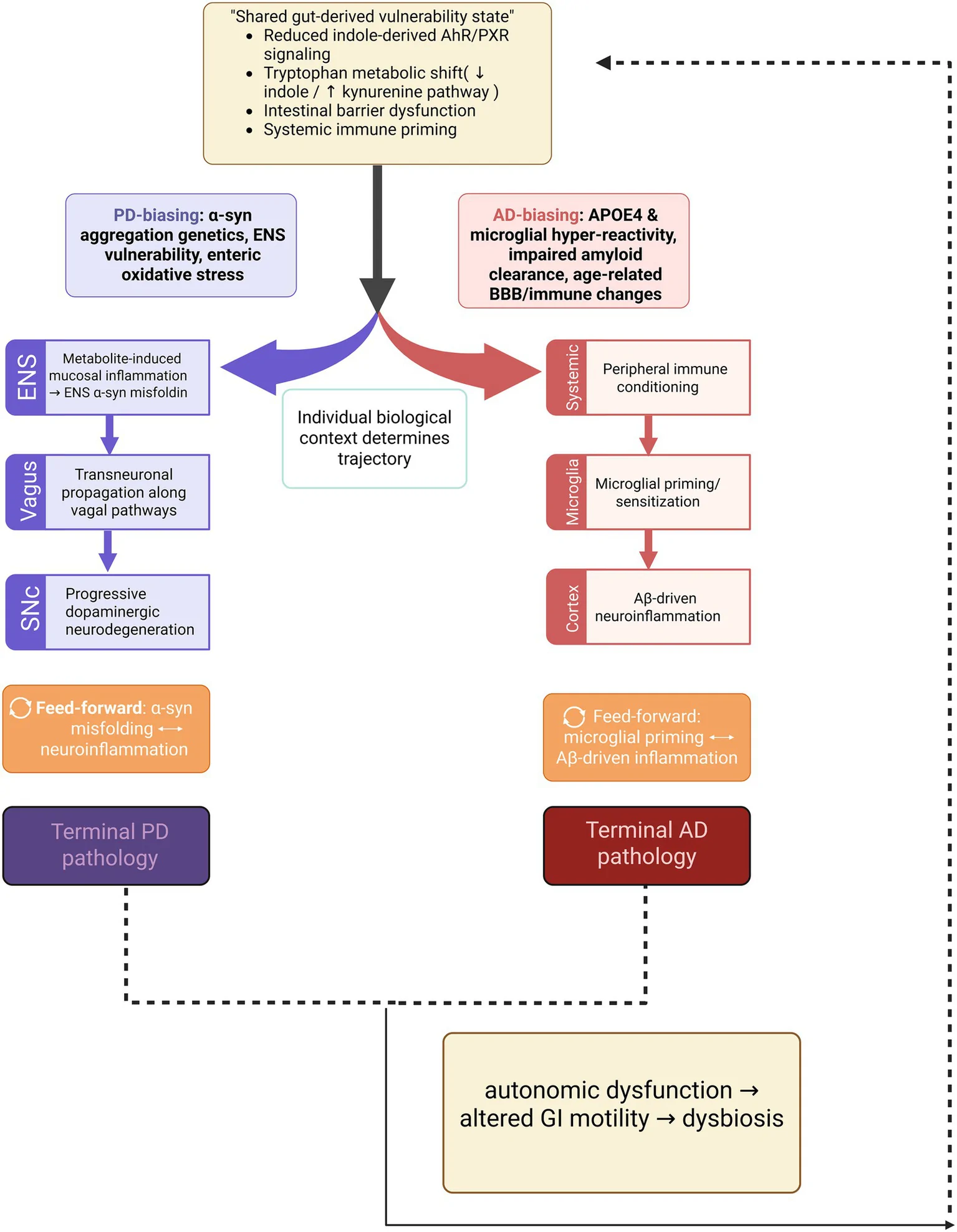
Proposed context-dependent trajectories from shared gut-derived disturbances to PD- and AD-related pathology. The upper box summarises the proposed shared gut-derived vulnerability state, comprising altered tryptophan metabolism, reduced indole-associated receptor signalling, barrier dysfunction, and systemic immune priming. Individual genetic and cellular contexts may influence the relative contribution of overlapping neural, immune and vascular processes in both diseases; the branches do not establish pathway dominance or a measured genotype–metabolite interaction. The purple branch depicts enteric inflammation and α-synuclein misfolding, followed by propagation along vagal pathways and dopaminergic vulnerability in a proposed gut-first PD trajectory. The red branch depicts peripheral immune conditioning, microglial sensitisation, and Aβ-associated neuroinflammation relevant to AD. The flanking boxes identify candidate susceptibility factors, including the *APOE4*-associated context; they do not imply exclusive genetic assignment to either pathway. Coloured arrows indicate proposed forward relationships, and orange boxes highlight reinforcing interactions between protein pathology and inflammation. Black dashed return arrows and the lower box depict potential feedback from CNS disease through autonomic dysfunction and altered GI motility to dysbiosis. These trajectories are hypotheses; their full sequence and temporal order have not been established prospectively in humans. PD, Parkinson’s disease; AD, Alzheimer’s disease; α-syn, α-synuclein; Aβ, amyloid-β; ENS, enteric nervous system; SNc, substantia nigra pars compacta; BBB, blood–brain barrier; GI, gastrointestinal; CNS, central nervous system; AhR, aryl hydrocarbon receptor; PXR, pregnane X receptor; *APOE4*, apolipoprotein E ε4 variant context. Created with BioRender.com (Agreement number: PH29NLJBPV; https://BioRender.com/qy9bcp0).

Several experimentally supported observations constrain this hypothesis. A human cellular reporter study linked the *LRRK2* R1441C variant to impaired autophagic balance (Alegre-Abarrategui et al., 2009); it did not establish selective vulnerability of enteric neurons. In G2019S macrophages, altered lysosomal tubulation and antigen presentation accompanied increased inflammatory cytokine release, and LRRK2 inhibition or knockdown modified these responses (Wallings et al., 2023). Thus, *LRRK2*-associated susceptibility can involve myeloid cells as well as neuronal processes. Glucocerebrosidase, encoded by *GBA1*, and LRRK2 are both measurable in human monocytes; altered enzymatic readouts in idiopathic PD monocytes provide evidence of overlapping immune-cell involvement, not proof of a *GBA1* genotype–metabolite interaction (Wallings et al., 2022). *APOE* ε4 likewise cannot be assigned solely to systemic immune conditioning: APOE isoform experiments in mice implicated pericyte cyclophilin A signalling in blood–brain barrier injury (Bell et al., 2012), while human *APOE* ε4 carriers showed barrier abnormalities associated with cognitive decline (Montagne et al., 2020). These vascular findings complement reported glial effects (Fernandez et al., 2019). None of these studies demonstrates that a gut metabolite preferentially selects a neural or immune disease trajectory.

The proposed intermediate steps are conditional: altered luminal metabolites would first change epithelial or mucosal immune responses; local mediator release or circulating exposure could then stress lysosomal, lipid-handling or vascular functions; genotype-dependent reserve in each cell population might modify downstream protein handling and inflammatory responses. These links require direct testing. For example, DCA-induced NF-κB activity in colonic cells (Glinghammar et al., 2002) does not establish DCA-driven enteric neuronal injury or its interaction with LRRK2. Microbial-metabolite-dependent microglial control of astrocytes in experimental autoimmune encephalomyelitis (EAE), a mouse model relevant to multiple sclerosis (Rothhammer et al., 2018), supports communication between cell populations, but not the proposed genetic allocation of disease pathways. We therefore make no claim that *LRRK2*/*GBA1* preferentially impair enteric neurons over immune cells, that *APOE* ε4 preferentially affects peripheral immunity over neural or vascular functions, or that these variants act synergistically with one another. A discriminating experiment would compare isogenic risk-variant and corrected/control backgrounds separately for *LRRK2*, *GBA1* and *APOE*, each under vehicle and defined metabolite exposures with dose–response measurements. Enteric neurons, macrophages/microglia and neurovascular models should be studied in parallel, measuring lysosomal flux, α-synuclein handling, cytokine responses and barrier function as appropriate. Matched cocultures and conditioned-medium transfer, followed by receptor blockade or mediator neutralisation, could distinguish direct exposure from epithelial–immune relay effects. A genotype × exposure interaction, prespecified on an additive outcome scale, would test whether the combined effect differs from the sum of genotype and exposure effects. Separate main effects alone would support independent/additive mechanisms. A claim of preferential cellular susceptibility additionally requires a direct comparison of exposure effects across cell types, with matched viability and exposure controls; genetic correction and pathway-specific rescue would help test the proposed intermediate steps.

Differences in cellular response thresholds are another proposed modifier of overlapping relay pathways; disease-specific thresholds have not been established. In biochemical experiments, 4-hydroxy-2-nonenal modification of α-synuclein inhibited fibrillation while promoting stable soluble oligomers that were toxic to primary mesencephalic cultures (Qin et al., 2007). This does not establish dysbiosis-induced production, an enteric-neuron threshold effect, or accelerated fibril formation. Any contribution to enteric vulnerability remains a hypothesis. Peripheral monocyte training and persistent changes in resident microglia should be considered separately. Trained immunity describes altered innate responses after an initial stimulus (Netea et al., 2020). In experiments with human monocytes, *Candida albicans* or β-glucan exposure enhanced subsequent cytokine responses and was associated with H3K4 trimethylation changes (Quintin et al., 2012); these findings do not establish the same response to chronic gut-derived LPS or metabolite exposure. Peripheral immune-cell entry into the CNS is context-dependent (Prinz and Priller, 2017), and evidence of monocyte recruitment does not itself demonstrate persistent reprogramming of resident microglia. In mice, peripheral inflammatory stimulation induced training or tolerance and lasting epigenetic changes in resident microglia; the stimulation regimen also altered amyloid pathology in an APP-transgenic model (Wendeln et al., 2018). This supports experimental peripheral-to-central immune conditioning, but does not establish a gut-monocyte migration pathway that transfers a trained chromatin state to microglia in human PD or AD. Whether chronic gut-derived signals produce such lasting microglial changes remains a hypothesis requiring cell-resolved and lineage-tracing experiments. These mechanisms may contribute to relative pathway susceptibility without defining an exclusive neural or immune route.

## 5 Therapeutic implications of the metabolite-driven framework

Translation requires mechanism-specific effects, adequate exposure, chronic safety and clinical benefit (Rob et al., 2025). Although EAE studies and multiple-sclerosis-related observations support tryptophan/AhR responses in astrocytes and microglial–astrocyte communication (Rothhammer et al., 2018; Rothhammer et al., 2016), broader neurological literature (Cryan et al., 2020; Rob et al., 2025) does not establish equivalent metabolite signatures or a universal ENS-first sequence across disorders. The following assessment therefore focuses on PD/AD treatments, biomarkers and timing.

Selected PD/AD metabolite and barrier studies are summarised in Table 1 (Section 2.2); the additional disorders discussed here are not covered by that table.

### 5.1 Therapeutic candidates and translational evidence

Microbiota-directed interventions and receptor-directed compounds differ in their evidence and specificity. A randomised PD probiotic trial reported improvement in constipation outcomes, without establishing disease modification (Tan et al., 2021). FMT has progressed beyond feasibility studies: GUT-PARFECT, a phase 2 trial involving 46 treated participants, reported a motor signal at 12 months (Bruggeman et al., 2024), whereas a separate Finnish trial randomising 48 participants found no benefit on its primary clinical outcome at 6 months (Scheperjans et al., 2024). A later phase 2 trial randomised 72 drug-naïve PD participants to repeated donor or autologous FMT; 66 completed follow-up. At 35 weeks, donor FMT improved motor and constipation scores relative to autologous FMT, and no serious treatment-related adverse events were reported (Zhang et al., 2026). This selected trial extends the symptomatic evidence but does not resolve differences among protocols or establish long-term safety, receptor-specific action or slowing of neurodegeneration. Taxonomic changes alone cannot demonstrate restoration of a particular metabolite pathway or receptor response.

Translation requires target engagement, mechanism-specific effects, pharmacokinetics, drug-interaction assessment, chronic safety and efficacy in the intended population. Table 2 distinguishes measured findings from proposed mechanisms. Exposure and pharmacodynamic responses must be linked to clinically meaningful benefit; biological plausibility or biomarker change alone does not establish readiness for long-term treatment or prevention.

**Table 2:** Therapeutic candidates, proposed mechanisms, measured evidence, and requirements for translation.

Target/strategy	Pathway/target	Proposed mechanism	Measured evidence	Evidence still required
AhR ligands	AhR in epithelial and immune/glial contexts	Modulate mucosal cytokine and barrier responses; effects may differ by ligand, tissue, species, dose and duration.	Experimental: indole–AhR/IL-22 mucosal responses (Zelante et al., 2013); astrocyte/microglial pathways in EAE mouse models relevant to multiple sclerosis (Rothhammer et al., 2016, 2018). These are not clinical efficacy studies.	Tissue target engagement and receptor dependence; exposure and enzyme/transporter effects; chronic immune safety; PD/AD efficacy.
IPA-based approaches	PXR–TLR4; ligand context	Modulate enterocyte inflammatory and barrier responses.	Experimental: IPA in an indole context engaged PXR; TLR4 loss corrected barrier defects in PXR-deficient mice (Venkatesh et al., 2014).	Human receptor-specific effects; oral stability, intestinal/systemic exposure and dose; interactions and chronic safety; relevant-population efficacy.
Rifaximin	Bacterial RNA polymerase; human PXR	Antibacterial changes and/or epithelial PXR signalling may alter barrier and inflammatory outcomes.	Experimental: PXR target-gene induction in humanised mice (Ma et al., 2007). Human AD pilot: *n* = 10, open-label, 3 months; NfL decreased, cognition unchanged (Suhocki et al., 2022). No demonstration of PXR mediation or disease modification.	Separate antibiotic and PXR effects; assess hepatic/comedication exposure and *C. difficile* risk (Salix Pharmaceuticals, 2023); resistance and ecological recovery (Turner et al., 2024); controlled neurological efficacy and chronic safety.
Butyrate / delivery formulations	HDACs; SCFA receptors including GPR109A	Potential epithelial, immune and proteostatic effects; contributions require separation.	Mouse: sodium butyrate in MPTP (Guo et al., 2023); GPR109A-dependent intestinal responses (Singh et al., 2014). Human PD: *n* = 72, 6-month SCFA/prebiotic trial; motor improvement, but all arms active and receptor mediation unproven (Hegelmaier et al., 2026).	Inactive-controlled replication; distinguish butyrate, propionate and prebiotic contributions; receptor-versus-HDAC effects; tissue exposure, interactions and chronic safety; disease-modifying efficacy.
Bile acid approaches, including UDCA	UDCA mitochondrial rationale; TGR5/FXR are distinct candidate targets	Potential bioenergetic or receptor-mediated effects; not interchangeable mechanisms.	Human PD: UDCA phase 2, *n* = 30, 48 weeks; exploratory bioenergetic signal, no MDS-UPDRS-III difference (Payne et al., 2023). Bile-acid associations (MahmoudianDehkordi et al., 2019; Nho et al., 2019) are not treatment evidence.	Confirm mechanism and exposure; long-term hepatic/metabolic safety and interactions; adequately powered efficacy trials; no inference of gut receptor engagement from UDCA results.
Probiotics, prebiotics and FMT	Indirect microbial/metabolic effects	Alter community function; restoration of specific indole/SCFA pathways requires measurement.	PD: probiotic constipation benefit (Tan et al., 2021); FMT motor signal (Bruggeman et al., 2024) and separate negative primary outcome (Scheperjans et al., 2024). Later drug-naïve trial: *n* = 72 randomised, donor vs. autologous FMT, 35-week motor/GI benefit (Zhang et al., 2026). No established disease modification.	Strain/donor specificity; reproducible metabolite and host responses; pathogen/long-term ecological safety; medication confounding; replicated clinical benefit.
IDO1 inhibitors	IDO1 enzyme; kynurenine pathway	Reduce IDO1-dependent flux; downstream CNS effects remain to be demonstrated.	Human oncology: epacadostat plus pembrolizumab, *n* = 706; no survival benefit in melanoma (Long et al., 2019). Not PD/AD or prevention evidence.	Tissue engagement versus TDO2; PK and comedication; long-term immune/infection safety. Combination-oncology tolerability cannot establish isolated IDO1-inhibitor prevention safety.
Sodium oligomannate (GV-971)	Proposed microbiota–metabolite–immune pathway	Mouse microbiota/amino-acid/Th1 mechanism (Wang et al., 2019); clinical mediation unproven.	AD trial: *n* = 818, 36 weeks; ADAS-Cog12–2.15 points; secondary endpoints nonsignificant (Xiao et al., 2021). 2025 disclosure: certificate expiry and suspended production/sales (Shanghai Fosun Pharmaceutical (Group) Co., Ltd., 2025). Aug 2026 update: confirmatory trial still enrolling (Shanghai Fosun Pharmaceutical (Group) Co., Ltd., 2026).	Confirmatory efficacy and regulatory reassessment; mechanism-linked exposure; human interaction studies, organ-impairment PK and chronic safety. Cognitive change alone does not establish disease modification.

Evidence refers to the cited studies and is labelled by model or clinical population. Receptor/metabolic mechanisms are proposed unless receptor dependence or another mechanistic test is specified; target engagement, symptomatic improvement and disease modification are distinct outcomes. Required evidence spans target engagement, mechanism specificity, pharmacokinetics, drug interactions, chronic safety and efficacy in the intended population. Selected therapeutic evidence was updated during revision; company disclosures are dated separately and are not efficacy studies. Registration, enrolment, symptom benefit, biomarker change and disease modification must not be equated. AhR, aryl hydrocarbon receptor; ADAS-Cog12, 12-item cognitive subscale of the Alzheimer’s Disease Assessment Scale; FMT, faecal microbiota transplantation; FXR, farnesoid X receptor; HDAC, histone deacetylase; IBS-D, diarrhoea-predominant irritable bowel syndrome; IDO1, indoleamine 2,3-dioxygenase 1; IPA, indole-3-propionic acid; MDS-UPDRS-III, Movement Disorder Society Unified Parkinson’s Disease Rating Scale motor examination; MPTP, 1-methyl-4-phenyl-1,2,3,6-tetrahydropyridine; PXR, pregnane X receptor; SCFA, short-chain fatty acid; TGR5, Takeda G protein-coupled receptor 5; UDCA, ursodeoxycholic acid.

AhR-associated mucosal IL-22 responses (Zelante et al., 2013) and astrocytic/microglial findings in EAE and multiple-sclerosis-related research (Rothhammer et al., 2016, 2018) are context-specific. Ligand, dose, duration, species and cell type matter; intestinal activation is not uniformly beneficial or CNS/systemic activation uniformly harmful. Gut-directed delivery requires measured exposure and receptor-dependent effects, followed by barrier or neural benefit at tolerable doses. Clinical disease modification in PD/AD remains unestablished.

For PXR, IPA-associated barrier effects in mice depended on the indole context and PXR–TLR4 signalling (Venkatesh et al., 2014); they do not establish uniformly anti-inflammatory pharmacology across ligands or species. Rifaximin is both an antibiotic and a human PXR ligand. Intestinal PXR target-gene induction was demonstrated in PXR-humanised mice, but not wild-type or *Pxr*-null mice (Ma et al., 2007). Clinical barrier or inflammatory changes could therefore reflect antimicrobial effects, receptor engagement, or both. Mechanistic studies should compare rifaximin with an antibacterial comparator lacking PXR activity and a non-antibacterial PXR ligand, together with receptor-deficient or humanised models. Sterile epithelial cultures can test direct receptor-dependent responses; defined-microbiota cocultures and paired microbial, metabolite and permeability measurements can test bacterial contributions while matching exposure and antibacterial activity. Rifaximin has established indications including reduction of recurrent hepatic encephalopathy and diarrhoea-predominant irritable bowel syndrome (Salix Pharmaceuticals, 2023), but experience in those populations does not establish neurological efficacy or prevention suitability. Repeated antibiotic exposure also requires ecological assessment. Experimental and clinical-isolate work demonstrated rifaximin-associated selection of daptomycin cross-resistance in vancomycin-resistant *Enterococcus faecium* (Turner et al., 2024); the risk magnitude in PD/AD populations remains unknown. Longitudinal culture/susceptibility testing, resistome analysis and metabolite profiling should assess selection of resistant organisms, loss or redistribution of microbial functions, and recovery after withdrawal. A small open-label AD pilot treated ten participants for 3 months: serum NfL decreased and microbiota composition changed, but cognition did not significantly improve (Suhocki et al., 2022). Without a placebo group or receptor-specific intervention, the study cannot attribute the biomarker change to PXR, establish neuroprotection or separate antibiotic and receptor effects. Its size and duration cannot resolve long-term safety. A favourable short-term symptom or barrier response would not resolve these questions.

Butyrate provides another experimental candidate, with several possible mechanisms. Sodium butyrate improved neuroinflammatory and neuronal outcomes in an MPTP mouse study (Guo et al., 2023), and GPR109A-dependent epithelial and immune effects were demonstrated in separate intestinal models (Singh et al., 2014). These results should not be conflated with FMT effects in MPTP mice (Sun et al., 2018), nor treated as proof of α-synuclein disease modification. HDAC inhibition, receptor signalling and indirect microbiota effects require separate perturbation experiments. A randomised, blinded six-month PD study enrolled 72 participants into propionate/butyrate, 2′-fucosyllactose, or combined supplementation arms and reported motor improvement (Hegelmaier et al., 2026). Every arm received an active intervention; matching placebo capsules did not create an inactive control group. Retrospective registration, descriptive analyses without multiplicity correction, concomitant PD treatment and short follow-up limit causal and disease-modifying interpretation. The study does not isolate a butyrate-specific receptor effect. Formulations intended for colonic or systemic delivery still require measured release, exposure–response and interaction data.

TFEB manipulation reduced α-synuclein toxicity (Decressac et al., 2013) and enhanced astroglial tau handling (Martini-Stoica et al., 2018) in separate models. Together with butyrate’s HDAC inhibition (Davie, 2003), these findings motivate, but do not establish, a butyrate–HDAC–TFEB treatment mechanism. Target engagement, TFEB-dependent rescue and protein-clearance changes must be demonstrated within the same relevant model.

Pharmacokinetics, interactions and chronic safety must be evaluated alongside target engagement. AhR/PXR studies should measure intestinal and systemic exposure over time, species-specific receptor activity, and effects on drug-metabolising enzymes and transporters, including candidate CYP1A1, CYP3A4 and P-glycoprotein readouts. The relevance of each change to concomitant medications requires clinical testing, particularly in older adults receiving multiple drugs. Rifaximin illustrates why low absorption is not sufficient reassurance: its prescribing information identifies increased systemic exposure with hepatic impairment and with P-glycoprotein inhibitors such as cyclosporine, and reports altered INR with concomitant warfarin (Salix Pharmaceuticals, 2023). Conversely, clinically significant CYP3A4 induction is not expected at recommended doses in people with normal liver function (Salix Pharmaceuticals, 2023); a receptor-based interaction hypothesis should not be presented as a demonstrated interaction in every patient. Candidate-specific studies should establish dose–exposure–response relationships, interaction liability under realistic comedication, and sustained gastrointestinal, hepatic and immune safety. The rifaximin label also warns of Clostridioides difficile-associated diarrhoea following antibacterial exposure (Salix Pharmaceuticals, 2023). Chronic antibiotic strategies therefore require infection and ecological surveillance, not only routine adverse-event counts. Safety over a short treatment course or in a different indication cannot establish a favourable risk–benefit balance for years of preventive exposure.

IDO1 inhibition targets an enzyme, not a receptor. In the 706-participant ECHO-301/KEYNOTE-252 melanoma trial, adding epacadostat to pembrolizumab improved neither progression-free nor overall survival (Long et al., 2019). Both arms received pembrolizumab, so the results cannot establish isolated IDO1-inhibitor safety during long-term prevention in neurologically asymptomatic older adults. PD/AD development requires tissue-specific engagement, distinction from TDO2 contributions, linkage of peripheral changes to intended CNS responses, pharmacokinetic and comedication assessment, chronic immune/infection safety and stage-specific clinical efficacy. Oncology exposure does not establish readiness for repurposing.

Sodium oligomannate (GV-971) requires separate appraisal of preclinical mechanism, clinical outcomes and regulatory context. Mouse experiments linked treatment to microbiota and amino-acid changes with reduced Th1-associated neuroinflammation (Wang et al., 2019); this does not establish the mechanism mediating benefit in humans. A Chinese phase 3 trial randomised 818 participants with mild-to-moderate AD to GV-971 (900 mg/day) or placebo for 36 weeks (Xiao et al., 2021). The ADAS-Cog12 treatment difference was −2.15 points (95% CI − 3.07 to −1.23), favouring GV-971, but prespecified global, daily-function and neuropsychiatric secondary outcomes were not significantly different. Reported adverse-event incidence was similar between groups over that interval (Xiao et al., 2021). This cognitive result does not by itself demonstrate slowed underlying disease progression, prevention, or a microbiota-mediated clinical effect. The historical Chinese indication was mild-to-moderate AD (Shanghai Green Valley Pharmaceuticals, 2021). A company regulatory announcement dated 22 December 2025 states that the conditional registration certificate expired in November 2024 and commercial production and sales were suspended at the announcement date; resumption required confirmatory-trial completion and regulatory review/re-registration (Shanghai Fosun Pharmaceutical (Group) Co., Ltd., 2025). A subsequent company update dated 25 August 2026 described the confirmatory trial as ongoing, reporting more than 1,000 participants enrolled by 31 July 2026 (Shanghai Fosun Pharmaceutical (Group) Co., Ltd., 2026). This is an enrolment update, not peer-reviewed efficacy data or confirmation of renewed authorisation. Neither disclosure establishes the final regulatory outcome at the time of reading. Historical authorisation should not be extrapolated to PD, prevention, disease modification or validated AhR/PXR modulation. The 2021 package insert reports no human drug-interaction studies and no pharmacokinetic studies in hepatic or renal impairment (Shanghai Green Valley Pharmaceuticals, 2021). Further evaluation should connect intestinal/systemic exposure to measured microbial and metabolic changes, test whether those changes mediate clinical outcomes, characterise interactions and chronic safety in older patients, and seek independent replication with clinical and disease-biology endpoints.

A 48-week phase 2 UDCA study in 30 PD participants primarily assessed safety and tolerability, with exploratory midbrain bioenergetic findings; it established neither motor efficacy nor a gut TGR5/FXR mechanism (Payne et al., 2023). Neural, immune and vascular outcomes can overlap in both diseases (Monroe et al., 2026), so treatment selection should not assume fixed pathway assignments. Prevention additionally requires validated enrichment and stage-specific benefit (Section 5.3).

### 5.2 Candidate biomarkers: intended uses and validation

Metabolomics can characterise peripheral small-molecule profiles (Wishart, 2019), but a patient–control difference is not diagnostic or predictive validation. Here, the primary proposed use of the IPA–Kyn/Trp–LBP combination is research phenotyping of gut-associated metabolic and barrier abnormalities, with exploratory trial stratification. It is not a validated test for the hypothesised vulnerability state. Prediction of disease onset, PD-versus-AD classification and monitoring treatment response are distinct secondary hypotheses, each requiring its own endpoint and validation. The candidate evidence below should be read by specimen, disease stage and intended use.

For research phenotyping, validation should compare the candidate combination with prespecified functional permeability and mucosal or immune measurements; none of its components directly measures epithelial AhR/PXR activity. Onset prediction requires longitudinal follow-up of initially unaffected or prodromal individuals and a defined prediction horizon. PD-versus-AD classification requires a direct differential-diagnosis cohort, including alternative diagnoses. Response monitoring requires repeated measurements in controlled intervention studies, showing reproducible changes linked to target engagement and clinical outcomes. Case–control AUCs cannot substitute for these evaluations.

#### 5.2.1 IPA

Li et al. (2025) measured serum and faecal IPA in controls, MCI and AD (Fig. 5: *n* = 9–11 per group), finding lower concentrations in the impaired groups. The human analysis did not report diagnostic AUC, sensitivity/specificity or independent validation of an IPA classifier; experimental multi-omics cross-validation in mice is not human diagnostic validation. Chen et al. (2022) studied fasting plasma in 56 established PD cases and 43 matched controls; mean PD duration was 5.0 years and mean off-medication Hoehn–Yahr stage was 2.3. IPA was higher in PD. An age/sex-plus-IPA model yielded AUC 0.66 (95% CI 0.54–0.77) in that same cohort. This is neither an IPA-only estimate nor an independently validated prodromal predictor; no externally validated sensitivity/specificity threshold was established.

#### 5.2.2 SCFAs

Unger et al. (2016) compared faecal measurements in 34 diagnosed PD patients and 34 controls; reduced concentrations were reported, without a validated AUC or sensitivity/specificity threshold for prodromal prediction. The microbiota meta-analysis addressed taxa rather than a direct SCFA diagnostic assay (Nishiwaki et al., 2020). In the plasma study, 28 participants had AD-related cognitive impairment (19 MCI, 9 dementia), 29 had non-AD impairment (19 MCI, 10 dementia), and 10 were cognitively unimpaired. Valerate/butyrate classified AD-related impairment versus unimpaired controls with AUC 0.918 (95% CI 0.830–1.000); acetate classified AD-related versus non-AD impairment with AUC 0.954 (0.901–1.000) (Marizzoni et al., 2025). These are same-cohort, cross-sectional estimates from small diagnostic groups, without independent external validation or a validated sensitivity/specificity threshold for screening. They do not establish prediction before symptoms or CNS pathology.

#### 5.2.3 Bile acids

The ADNI serum analysis included 1,464 participants (370 cognitively normal, 284 early MCI, 505 late MCI and 305 AD). Higher DCA:CA was associated with worse cognition; association-level replication in the independent ROS/MAP resource should be distinguished from validation of a diagnostic model. In 789 MCI participants, baseline CA and GDCA:CA/TDCA:CA were associated with later AD dementia progression; 251 converted and 538 did not within 4 years (MahmoudianDehkordi et al., 2019). The cited analyses do not provide an externally validated clinical prediction model with AUC, sensitivity/specificity and calibrated risk thresholds for this intended use. The separate ADNI pathology-biomarker analysis (*n* = 1,562, endpoint-specific subsets) relates serum bile acids to CSF/imaging outcomes (Nho et al., 2019); it is not an independent cohort replication or proof of metabolite change before CNS pathology.

#### 5.2.4 Kynurenine measures

Paired serum/CSF analyses in 33 PD patients, 33 AD patients and 39 controls found reduced CSF kynurenic acid and age-associated changes shared across groups (Sorgdrager et al., 2019). These were clinically diagnosed groups, not a prospective prodromal cohort; the study does not establish diagnostic AUC, sensitivity/specificity or independent validation for the proposed plasma Kyn/Trp component. Kyn/Trp and an indole-to-kynurenine ratio are different candidate measurements. The latter has no fixed analyte definition, validated threshold or diagnostic performance in the evidence reviewed here, and neither ratio directly measures receptor activity or microbial–host substrate competition.

#### 5.2.5 Barrier-associated measures

In nine early PD patients and ten controls, median PD duration was 2 years and median Hoehn–Yahr stage was 2. Plasma LBP was lower in PD; urinary sucralose recovery increased, whereas lactulose/mannitol did not (Forsyth et al., 2011). No diagnostic AUC, sensitivity/specificity threshold or external validation for identifying the proposed vulnerability state was established. Plasma zonulin was studied in 71 mild AD cases, 66 moderate AD cases and 74 controls (Qaisar et al., 2023). Its reported ROC analyses concern poor postural balance in AD, not AD diagnosis or future onset; those endpoints cannot supply performance estimates for neurodegeneration screening. Independent validation for the proposed gut-phenotyping use was not established. Accordingly, neither an elevated-LBP rule nor zonulin alone defines the proposed state.

Established biomarkers answer different biological questions. Plasma NfL reflects neuroaxonal injury rather than gut dysfunction or a specific proteinopathy. Ashton et al. (2021) studied two multicentre cohorts (KCL *n* = 805; BioFINDER *n* = 1,464), including cognitively unimpaired participants and multiple neurological diagnoses; AD dementia versus amyloid-negative unimpaired classification gave AUCs of 0.79 and 0.80, respectively, with age- and diagnosis-dependent performance. This two-cohort validation does not validate the gut panel. Plasma Aβ42/40 addresses brain amyloidosis: in 158 mostly cognitively normal participants, its amyloid-PET AUC was 0.88 (95% CI 0.82–0.93), increasing to 0.94 (0.90–0.97) with age and *APOE* ε4 status (Schindler et al., 2019). These estimates concern PET status in that study, not an independent test of the proposed gut combination. CSF amyloid measures and PET similarly address AD biology (Jack et al., 2018). CSF *α*-synuclein SAA detects seeding activity: a multicentre PPMI analysis included 1,123 participants, with 545 PD cases, 163 controls, 51 prodromal participants and other at-risk groups. PD-versus-control sensitivity was 87.7% (95% CI 84.9–90.5) and specificity 96.3% (93.4–99.2); positivity varied by genotype and olfaction. Prodromal positivity was not a longitudinal conversion-performance estimate (Siderowf et al., 2023). These assays therefore complement different research questions and should not be treated as interchangeable benchmarks.

No direct comparison or incremental-value analysis of the proposed IPA–Kyn/Trp–LBP combination against NfL, plasma Aβ42/40 or CSF *α*-synuclein SAA is established by the cited evidence. The SCFA study compared individual AUCs with neural/glial markers (Marizzoni et al., 2025), but did not test whether adding this gut combination improves a prespecified clinical model. The combination itself has no fitted and externally validated model, sensitivity/specificity threshold, AUC or demonstrated clinical utility. A future study should compare a clinical-covariate model plus the biomarker appropriate to the endpoint with the same model plus the gut measurements, using held-out external data, changes in discrimination with confidence intervals, calibration and clinical net benefit. Research phenotyping requires validation against gut endpoints before any disease-prediction claim.

Diet, medication, age, sampling time, matrix and analytical platform should be prespecified and harmonised; repeated sampling should assess within-person variability. Established-disease AUCs may not generalise to prodromal populations, but their degree or direction of bias cannot be assumed. Blood or stool sampling may facilitate repeated collection (Wishart, 2019); practical accessibility does not establish superiority over existing blood biomarkers, reproducibility, cost-effectiveness or clinical utility. Until the intended uses and external performance are validated, these candidates remain research measurements.

### 5.3 Timing and the proposed gut-derived intervention window

An optimal timing for gut-targeted disease modification is unknown. Constipation and idiopathic RBD provide clinical staging information (Savica et al., 2009; Postuma et al., 2019), but do not locate the proposed metabolic state relative to CNS pathology or establish its reversibility.

Constipation was associated with later PD even when documented at least 20 years before motor onset (Savica et al., 2009). In 1,280 people with polysomnography-confirmed idiopathic RBD, phenoconversion was estimated at 6.3% annually and 73.5% by 12 years (Postuma et al., 2019). These are symptom-to-diagnosis observations, not serial metabolite or pathology measurements. Prediagnostic gastrointestinal symptoms, prodromal metabolite alterations and metabolite changes preceding CNS pathology are distinct evidence categories. Testing the third requires repeated metabolite and pathology measurements in the same individuals; neither symptoms nor clinical conversion establish a metabolite-specific intervention window.

MCI likewise supplies a clinical staging anchor. Altered bile-acid profiles (MahmoudianDehkordi et al., 2019) and lower IPA (Li et al., 2025) during MCI do not establish changes before amyloid pathology. Baseline serum bile acids in 789 MCI participants were associated with subsequent AD dementia progression over 4 years (MahmoudianDehkordi et al., 2019), providing clinical temporal ordering rather than a pre-CNS therapeutic window. Detectability before MCI, including in cognitively normal *APOE* ε4 carriers, requires prospective study.

RBD and MCI may support enriched trial recruitment, but neither is established to lie within a metabolite-relevant treatment window. Earlier intervention benefit must be tested, with sufficient follow-up, sample size and validated intermediate endpoints.

AD frameworks and PD clinical literature (Jack et al., 2018; Kalia and Lang, 2015) do not establish loss of gut-targeted efficacy after CNS pathology develops. Stage-stratified controlled trials must test relative responsiveness, reversibility and disease modification without assuming an optimal stage.

Prospective biomarker validation (Section 5.2) and stage-stratified trials should therefore proceed together. Prevention designs require validated entry criteria and an acceptable risk–benefit profile; the framework provides a rationale, not evidence of efficacy.

## 6 Future perspectives

The main research tasks are to resolve causal direction, test the proposed interactions and establish clinically meaningful responses to intervention.

### 6.1 Limitations and contradictory evidence

Brain-first PD models allow CNS pathology to spread through autonomic pathways and produce secondary gut changes (Borghammer and Van Den Berge, 2019; Borghammer, 2018). Absence of enteric Lewy pathology in some autopsy cases and unresolved gut–brain timing challenge a universal gut-first sequence. The models may describe different subgroups; this framework applies most directly to testing gut-first contributions and cannot exclude reverse causality.

Contradictory findings constrain generalisation. Lower faecal SCFAs in PD (Unger et al., 2016) and mixed plasma changes in AD-related impairment (Marizzoni et al., 2025) do not establish uniform depletion. Mouse microbiota-transfer, butyrate-related and tau findings (Zhang et al., 2023) do not establish the same patient sequence. Human vagotomy evidence remains confounded and statistically uncertain (Svensson et al., 2015; Liu et al., 2017). PD microbiome studies also vary in sampling and confounder control (Boertien et al., 2019), without systematically comparing PD and AD metabolite signatures. Stratified analyses, dietary standardisation and repeated sampling are needed to assess disease, matrix and within-person variation.

Priorities are prospective multi-omics cohorts for temporal ordering; complementary genetic and controlled perturbation designs for causal inference; factorial genotype–metabolite tests (Section 4.3); and trials using independently validated enrichment criteria. No single approach establishes the entire causal sequence.

Additional confounders and sampling limitations require attention. Age, sex, diet, genotype and medication should be recorded in metabolomic analyses; a mouse study demonstrated genetic and environmental influences on microbiota composition, not established sex-specific AhR effects in human neurodegeneration (Org et al., 2015). Human cohort analyses associated PPI use with microbiota differences (Imhann et al., 2016); broader medication effects require drug-specific evidence rather than extrapolation from that PPI study. PD microbiome studies differ in sampling and confounder control (Boertien et al., 2019). Paired mucosal and luminal sampling in rhesus macaques showed location-dependent composition alongside substantial correlations with stool (Yasuda et al., 2015); this does not demonstrate selective underrepresentation of AhR/PXR-relevant bacteria in human PD/AD faecal samples.

### 6.2 Testable predictions of the framework

Two predictions test distinct components of the framework without presuming superiority over existing models.

#### 6.2.1 Prediction 1

The prespecified IPA–Kyn/Trp–LBP combination should improve independent measurement of gut-associated metabolic/barrier abnormalities beyond individual markers and clinical covariates (Section 5.2). Neurological prediction is a separate longitudinal extension requiring a defined endpoint, time horizon and comparison with endpoint-appropriate biomarkers in an independent cohort. PPMI or BioFINDER use depends on sample, assay and follow-up availability. Failure to improve out-of-sample performance would weaken the composite-marker prediction, not by itself disprove individual receptor mechanisms.

#### 6.2.2 Prediction 2

Genotype should modify the response to metabolite exposure beyond independent or additive effects. The factorial experiments in Section 4.3 test this separately for *LRRK2*, *GBA1* and *APOE* across neural, immune and vascular outcomes. Longitudinal cohorts should test prespecified genotype × metabolite terms with stage, treatment and confounder adjustment; significance within one subgroup alone is insufficient. Treatment-response modification requires a controlled genotype comparison. Adequately powered estimates consistent with additivity would weaken the interaction hypothesis while allowing separate effects. Precedence over CNS pathology remains a distinct temporal test.

## 7 Conclusion

Epithelial metabolite sensing offers a framework for connecting heterogeneous gut–brain findings while distinguishing human associations, experimental mechanisms and untested interactions.

A shared gut-derived vulnerability state could contribute to PD/AD in some individuals, but its co-occurrence, temporal precedence and reversibility remain unestablished. Neural, immune and vascular processes overlap; genotype-dependent susceptibility is a hypothesis rather than a fixed disease–pathway assignment. Composite gut phenotyping and genotype–metabolite interaction provide separate empirical tests. Longitudinal pathology measurements and controlled, stage-stratified interventions are additionally needed to determine causal direction and therapeutic value. Neither temporal precedence nor earlier treatment alone guarantees disease modification.

## Glossary

AD: Alzheimer’s disease
AhR: Aryl hydrocarbon receptor
*APOE4*: Apolipoprotein E ε4 allele
BBB: Blood–brain barrier
CNS: Central nervous system
DMV: Dorsal motor nucleus of the vagus
ENS: Enteric nervous system
FMT: Faecal microbiota transplantation
FXR: Farnesoid X receptor
GLP-1: Glucagon-like peptide-1
GPCR: G protein-coupled receptor
HDAC: Histone deacetylase
IAld: Indole-3-aldehyde
IAA: Indole-3-acetic acid
IDO1: Indoleamine 2,3-dioxygenase 1
IFN-γ: Interferon-gamma
IL: Interleukin
IPA: Indole-3-propionic acid
LPS: Lipopolysaccharide
MLCK: Myosin light chain kinase
MPTP: 1-methyl-4-phenyl-1,2,3,6-tetrahydropyridine
NF-κB: Nuclear factor kappa B
NTS: Nucleus tractus solitarius
PD: Parkinson’s disease
PXR: Pregnane X receptor
QUIN: Quinolinic acid
SCFA: Short-chain fatty acid
SNc: Substantia nigra pars compacta
STAT3: Signal transducer and activator of transcription 3
TDO: Tryptophan 2,3-dioxygenase
TGR5: G protein-coupled bile acid receptor 1 (GPBAR1)
TJ: Tight junction
TLR: Toll-like receptor
TNF-α: Tumour necrosis factor alpha
ZO-1: Zonula occludens-1
